# Cognitive dissonance in tuberculosis stigma: A mixed methods analysis of tuberculosis stigma measurement in South Africa

**DOI:** 10.1371/journal.pgph.0003932

**Published:** 2024-11-20

**Authors:** Alanna J. Bergman, Chakra Budhathoki, Michael V. Relf, Nkateko Ndlouvu, Nomusa Mthimkhulu, Sibongile Lerefolo, Kelly Lowensen, Jason E. Farley

**Affiliations:** 1 University of Virginia, School of Nursing, Charlottesville, Virginia, United States of America; 2 Center for Infectious Disease and Nursing Innovation, Johns Hopkins University, Baltimore, Maryland, United States of America; 3 School of Nursing, Johns Hopkins University, Baltimore, Maryland, United States of America; 4 School of Nursing, Duke University, Durham, North Carolina, United States of America; 5 Center for Infectious Disease and Nursing Innovation, Johns Hopkins University, Port Shepstone, Republic of South Africa; Federal University of Rio de Janeiro, BRAZIL

## Abstract

**Background:**

The Patient and Community Perspectives Towards Tuberculosis are the most common measure of tuberculosis (TB) stigma in sub-Saharan Africa. The instrument and its sub-scales (patient and community) have been quantitatively validated but have not undergone qualitative exploration in South Africa.

**Methods:**

We explored whether the Patient Perspectives Towards Tuberculosis adequately represents stigma as experienced by people with TB in South Africa. We used mixed methods to explore differences between the lived experience of TB and the quantitative stigma score. Participants with rifampicin-resistant TB and HIV co-infection completed the quantitative scale. Among those, 30 also completed qualitative interviews about their experiences and perceptions of TB stigma. We used cognitive interviewing techniques to interrogate congruence between the two data sources.

**Results:**

The scale demonstrated adequate factor structure with approximately normal distribution. Participants qualitatively described experiences and perceptions of stigma that contradicted their quantitative responses. The scale could not discriminate between participants who reported distressing experiences of TB stigma, and those who did not. Item wording caused confusion, and many elements of TB stigma most discussed by participants are not reflected in the scale.

**Conclusions:**

The Patient Perspectives Towards Tuberculosis lacks theoretical and experiential domains that are central to TB stigma in South Africa. Studies validating stigma scales in new populations should integrate a mixed-methods approach to ensure content validity.

## Background

Tuberculosis (TB) stigma disincentives people from engaging in TB testing, and treatment activities [1–5] and therefore is a major barrier to global TB elimination targets. Despite wide acknowledgement of stigma as a clinically significant barrier to care engagement, TB stigma is notoriously difficult to quantify [6, 7]. Stigma is a latent variable consisting of several underlying domains that elude direct measurement. Disease related stigma is also dependent upon socially constructed values which vary across geographic, cultural, and political strata [7, 8]. Due to the complexity of stigma measurement, many researchers use unvalidated questionnaires or proxy variables to capture the experience and breadth of TB stigma [7], however this contributes to poor scientific consensus about TB stigma measurement.

The Van Rie *Community and Patient Perspectives Towards Tuberculosis* are the most widely used validated instrument for measuring TB stigma [7]. The instrument has two separate sub-scales for measuring community-level and patient-level TB stigma respectively [9]. The scales were originally developed in Thailand; in-depth interviews and focus groups were conducted to evaluate representation, clarity, and content validity in a sample of Thai people with TB [8]. During the initial validation, authors performed an unrotated factor analysis suggesting two to three factors between all instrument items [9]. After three oblique rotations, two factors were identified and conceptually defined with one sub-scale per factor, (1) *community perspectives towards tuberculosis* and *(2) patient perspectives toward tuberculosis* [9]. The scales were later revalidated using factor analysis in various contexts throughout sub-Saharan Africa without additional qualitative exploration.

The *Community Perspectives towards Tuberculosis* are a collection of 11-items intended to measure how a status-neutral community perceives TB [9]. The Community Perspectives can be used among people with or without TB disease [9, 10]. The *Patient Perspectives Towards Tuberculosis* are intended to measure stigma among people with TB disease and include 12-items about disclosure, blame and transmission risk [9, 10]. The *Community Perspectives Towards Tuberculosis* have since been revalidated for cultural relevance throughout sub-Saharan Africa. Bresenham et al. psychometrically explored the scale in South Africa dropping three items due to poor performance [10]. They have since used the revised community items to measure TB stigma among community members, and people suspected of and diagnosed with TB [10]. A second revalidation study conducted in Lesotho explored the factor structure of the Community Perspectives among a sample of people with TB and HIV. The Lesotho team added three additional items intended to capture internalized stigma in this population [11]. In 2016, researchers performed factor analyses on both the *Community and Patient Perspectives Towards Tuberculosis* in Swaziland which demonstrated adequate structure, but did not employ other measures to assess construct validity [12].

Even with well validated measures, TB stigma remains dynamic, shifting in response to local disease burden, perceptions of infectiousness, and community perceptions of blame or control over infection. For example, in South Africa, where the burden of HIV is particularly high, TB and HIV stigma may have an exponential effect, compounding or intensifying stigma. The aforementioned factors may change with advances in prevention and treatment, and in response to political and public health messaging. As a result, complex constructs such as stigma are difficult to capture in a quantitative instrument that attempts to simplify and/or generalize a nuanced experience.

The purpose of this mixed methods study was to compare and contrast the lived experience of TB and TB stigma with quantitative stigma scores to evaluate whether the *Patient Perspectives Towards Tuberculosis* capture the depth and breadth of TB stigma among people with rifampicin-resistant TB (RR-TB) in South Africa’s epidemic.

## Methods

All research activities were conducted at a district TB hospital in KwaZulu-Natal (KZN) Province, South Africa. The local population is 89.9% Black African, with a poverty rate between 11–19% [13]. The study site is one of three district hospitals within Ugu District serving approximately 200,000 local individuals [13]. At 312 per 100,000 persons, Ugu District has one of the highest average rates of TB deaths in South Africa [13].

This was a nested cohort study, within a parent randomized clinical trial. In 2019 when the parent study was conceptualized, the South African TB guidelines endorsed modified directly observed therapy [14]. The parent study aimed to measure the effectiveness of an escalating community health worker adherence intervention offering enhanced support and lab monitoring triggered by video directly observed therapy while participants in the control arm received standard care without community health worker adherence supports. While the parent study had no aims towards stigma or stigma reduction participants did receive standardized health education regarding TB transmission and treatment which may help to reduced stigma (please see S1 Appendix). Participants eligible for the parent study were 18 years or older, tested positive for RR-TB and HIV, started on outpatient RR-TB treatment, reported access to cellular network at home, and reported literacy in either Afrikaans, English, isiXhosa or isiZulu. Everyone who presented to the outpatient TB ward was evaluated for eligibility. All participants enrolled in the parent study completed the *Patient Perspectives towards Tuberculosis Stigma Scale*. Local research assistants administered the scale in one of the four study languages using a standardized introduction, “Now, I am going to read a few statements about TB and HIV. These statements are meant to help us understand how you and others in the community think and feel about TB and HIV. These statements do not reflect how I personally feel about TB or HIV. Please tell me if you strongly agree, agree, disagree or strongly disagree with each of these statements.” The scale was administered at baseline, 3-months, 6-months and at study outcome. Scores from each timepoint were used for Pearson’s correlation. All other reported data is restricted to baseline only. Thirty participants from the parent study were also invited to participate in qualitative interviews to compare the lived experiences of TB stigma to the scores obtained from the quantitative data. Quantitative data were collected via direct entry into Research Electronic Data Capture (REDCap) which allowed for real time analysis.

The 12-item *Patient Perspectives towards Tuberculosis Stigma* Scale uses a 4-point Likert scale where 0 = strongly disagree, and 3 = strongly agree [9]. The items of the scale are summed and adjusted for interpretation on a 0–50 scale where zero is equivalent to little or no stigma, and 50 is associated with the highest levels of TB stigma [SS_50_ = (SS_raw_ × 50)/(*n* × 3)] [9]. There are no reverse scored items in the scale. The scale was used in its original form without adaptation or adjustment. The scale was professionally translated into all four study languages, with South African community health workers reviewing all items for clarity in local languages. Stigma items are available below in tables two and three. Stata version 16.0 was used to analyze the stigma data. Descriptive statistics were used to explore individual items and the scale as a whole. The mean (standard deviation) or median (interquartile range) distribution, or skewness, and evidence of floor and/or ceiling effects were of particular interest. An exploratory factor analysis was performed with the baseline scores; iterated principal-factor axis method (oblique rotation) was used to evaluate construct validity in the sample retaining one factor as conducted in the original study [9]. Because we considered the possibility of an intervention effect on stigma, we also used repeated measures Anova and generalized estimating equations to compare stigma scores over time to assess the instrument’s temporal stability.

Qualitative participants were initially enrolled consecutively. After successful recruitment of the first 10 participants, additional participants were enrolled purposively to maximize variability across gender, age, and baseline stigma score. Qualitative interviews were conducted by two experienced interviewers trained in qualitative methods, one was a Black South African woman who conducted all isiZulu interviews (n = 22), a Black US-based researcher conducted all English interviews (n = 8). Interviews ranged in length from 35 to 75 minutes and used probing questions to explore perceptions of self-concept and identity, TB-related stigma, status disclosure and supportive or coping factors. All interviews were audio recorded, deidentified and transcribed verbatim then uploaded into Atlas.ti for storage and organization.

The research team drew from existent stigma theory to develop codes and frame our interpretation. This included the seminal work of Link and Phelan. In their paper “Conceptualizing Stigma” Link and Phelan explore stigma not as a passive experience but as an exertion of, and response to, power. Their theory of stigma as a continuum from labeling through discrimination concludes that stigma and the conferral or divestment of power occurs in degrees across a broad spectrum of experience [8].

The first author read and reread the first ten interviews to develop a deep familiarity with the content considering similarities and divergent perspectives across participants. From these initial interviews the first author developed a codebook that was applied to the remaining transcripts. Some codes were generated a priori to reflect the domains of TB stigma as theorized in key prior literature [6, 15–27]. Interviews were coded initially by the study PI, and then reviewed by South African members of the research team to verify and contextualize codes and provide feedback. The team used thematic analysis to link and interpret codes. Authors employed the Standards for Reporting Qualitative Research checklist to increase transparency of our methods.

As researchers noted discordance between the qualitative and quantitative data, cognitive interviewing techniques were added into the interview guides to explore responses to the stigma questions [18]. Cognitive prompts included, “What is this question asking about?”, “why did you choose to answer the question this way?”, “I see that in the quantitative survey you choose this response, this seems different from how you explained your experience, can you tell me more?” Participants responded to the quantitative scale and then engaged in the interview. The interviewer would use the stored stigma scale to ask for reflections on their responses and expand upon answers. Authors employed the Standards for Reporting Qualitative Research checklist to increase transparency of our methods [19].

### Ethics

This study was approved by the Johns Hopkins University School of Medicine Institutional Review Board (IRB00211518) and the University of Witwatersrand Human Research Ethics Committee (M190937). Additionally, the hospital medical director and provincial and national directors of TB control were informed of the study and approved the protocol. All study participants provided written informed consent for the parent study and the qualitative interviews.

## Results

In total, 30 participants completed both qualitative and quantitative stigma assessments with additional participants completing the quantitative scale (n = 59). Consistent with epidemiologic TB studies, there were more male participants (58.8%) and mean age was 36.3. Among this sample in KZN, most spoke isiZulu at home (84.3%), were unemployed (62.8%), and were previously treated for TB (60.8%) (See Table 1). All participants had confirmed rifampicin-resistant TB (RR-TB) and HIV co-infection.

**Table 1:** Demographics.

Demographic Variable		Quantitative Sample (n = 59)	Qualitative Sample (n = 30)
		(n) %	(n) %
Randomization arm	Intervention	(29) 49.2%	(12) 40%
	Control	(30) 50.8%	(18) 60%
Sex	Male	(35) 59.3%	(19) 63.3%
	Female	(24) 40.7%	(11) 36.6%
Age	< 30	(8) 13.6%	(6) 20%
	30–39	(30) 50.8%	(15) 50%
	40–49	(14) 23.7	(6) 20%
	>50	(7) 11.9%	(3) 10%
Language spoken at home	English	(1) 1.7%	Interview language
	IsiXhosa	(7) 11.9%	English (8) 26%
	isiZulu	(51) 86.4%	IsiZulu (22) 73%
Employment status	Unemployed	(35) 59.3%	(17) 56.6%
	Part time	(7) 11.8%	(3) 10%
	Full time	(17) 28.9%	(10) 33.3%
Income*	<R 624	(18) 30.5%	(8) 26.67%
	R 624 –R 1334	(8) 13.6%	(4) 13.3%
	≥R1335	(33) 55.9%	(18) 60%
Prior TB treatment	Yes	(31) 60.8%	(20) 66.7%
	No	(20) 39.2%	(10) 33.3%
Time since HIV diagnosis	Median	6.4 Years	6.15 Years
	IQR	1.6–9.1 Years	3.3–8.7 Years
HIV viral suppression at baseline	HIV viral load ≥ 200	(22) 43.1%%	(15) 50%
	HIV viral load < 200	(26) 51.0%%	(15) 50%
	Missing	(3) 5.9%	n/a
Weight at baseline	Median	56 kg	54.5 kg
	IQR	51–61 kg	51–60.8 kg
BMI at baseline	Median	19.3	19.0
	IQR	18.6–21.5	18.2–20.9

*In 2021, R1,335 ($70.4) per month per person was the upper bound poverty limit in South Africa, R624 was the threshold for food poverty. TB, tuberculosis; HIV viral suppression, viral load <200 consistent with the South African antiretroviral therapy guidelines; Kg, kilogram; BMI, body mass index

The scale’s internal consistency reliability was good, Cronbach’s alpha = 0.91 at baseline with Pearson test-retest correlation of 0.72 (p<0.05) between baseline and month 3, 0.63 (p<0.05) between baseline and month 6 (see Table 2). Despite significant differences in Pearson correlation between the two timepoints, there were no statistically significant differences between mean stigma scores at each timepoint.

**Table 2:** Pearson correlation between stigma scores over time.

	Baseline TB Stigma	M3 TB Stigma	M6 TB Stigma
Baseline TB Stigma	1.000		
M3 TB Stigma	0.72 (p<0.001)	1.000	
M6 TB Stigma	0.51 (p<0.001)	0.63 (p<0.001)	1.000

**Fig 1:**
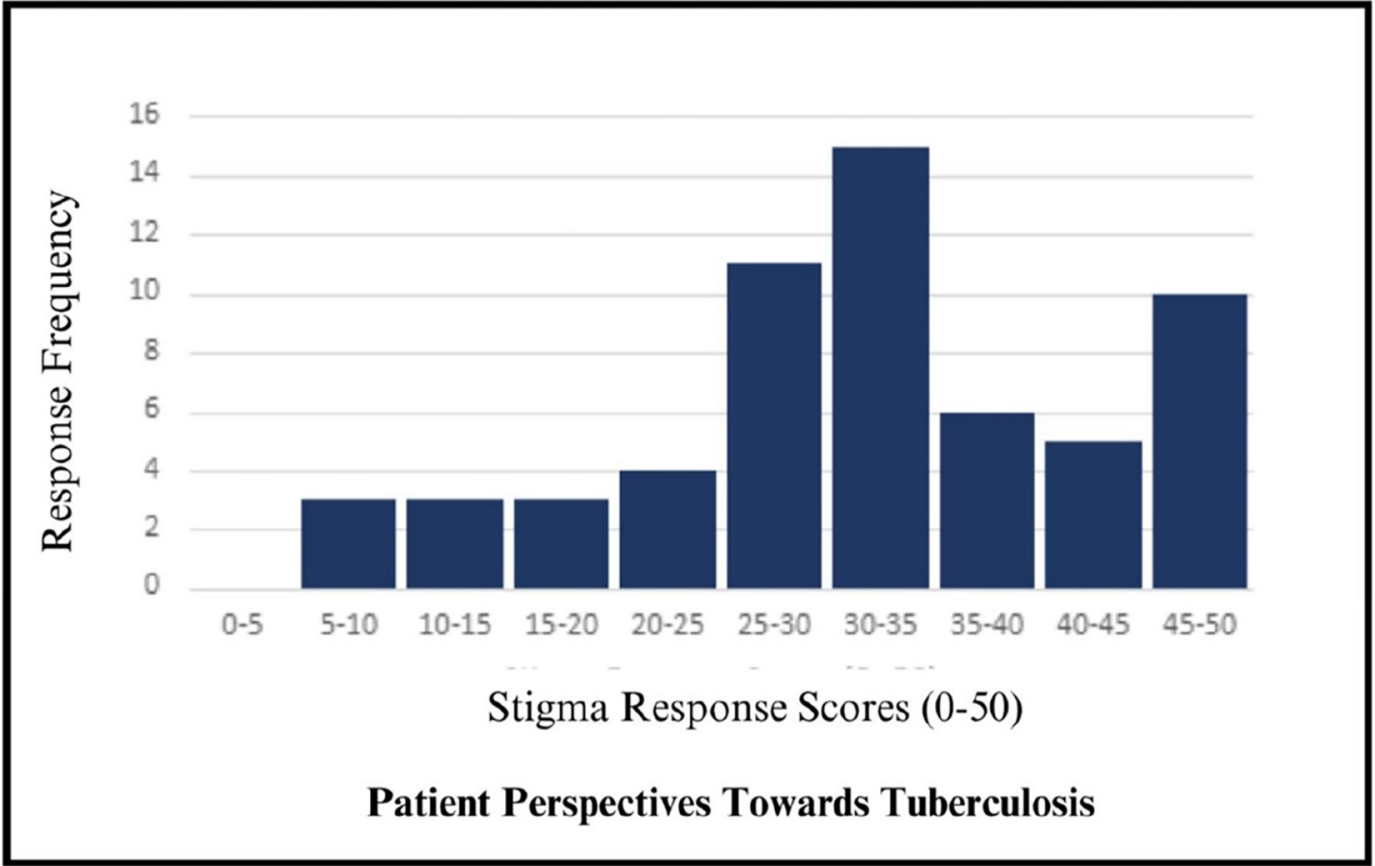
Distribution of corrected stigma scores.

**Fig 2:**
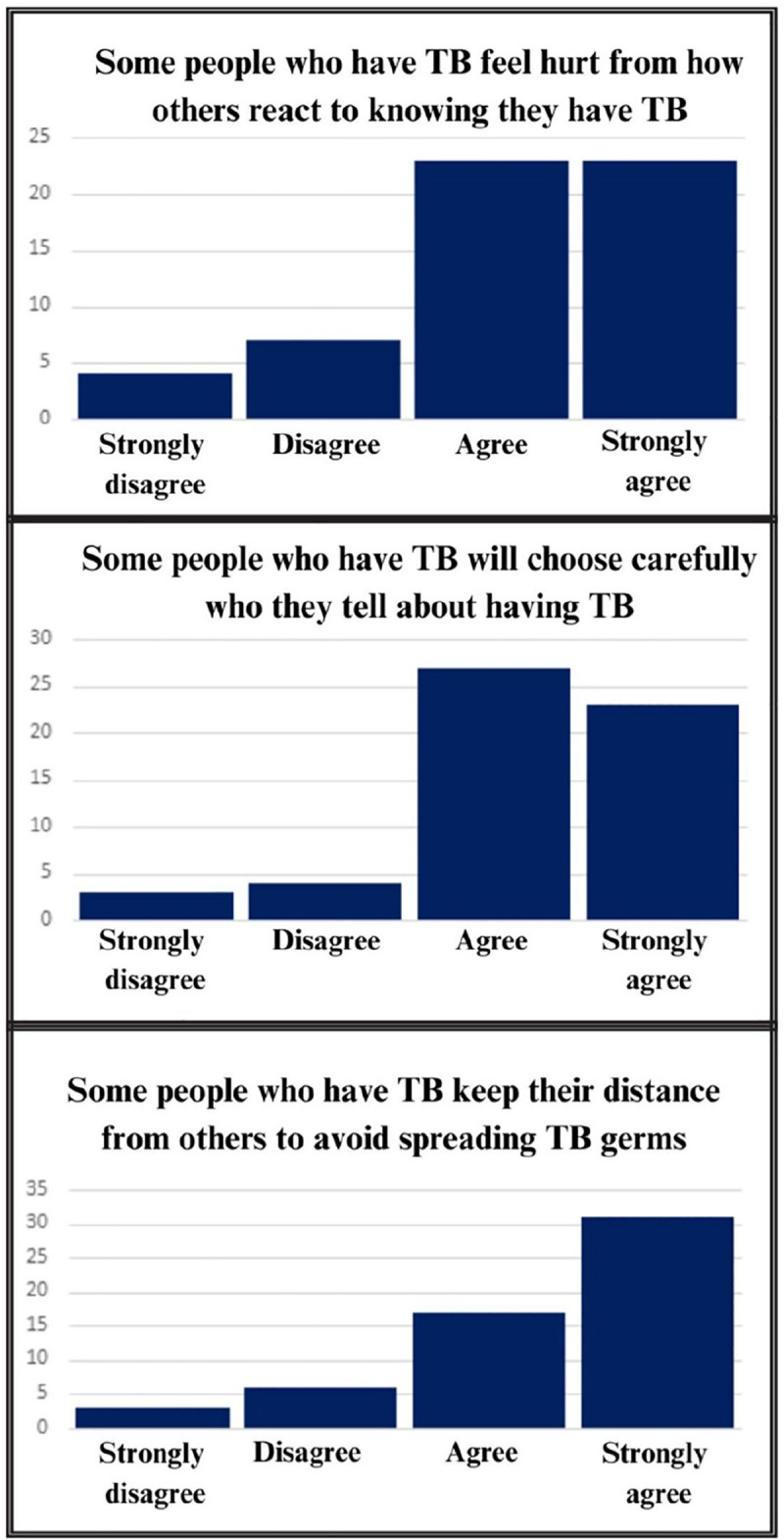
Instrument items with >80% endorsement.

Fig 1 shows the distribution of responses to the *Patient Perspectives Towards TB* at RR-TB treatment initiation. Scores are slightly skewed with most responses concentrated at the higher end of the scale (25–50). At baseline, median stigma score was 31.9 out of 50 (IQR 26.4–40.3). 5.3% of respondents scored in the lowest quintile (0–10) and 25% of respondents scored in the top quintile (score 40–50). 5.3% of participants scored the highest possible score, 50/50 on the scale while no participants scored below 5. Three items skewed very high with at least 80% of the sample agreeing or strongly agreeing with the statement (See Fig 2). These response distributions indicate limited variance within the sample on items related to disclosure, experienced stigma, and physical separation from others. Two additional questions regarding guilt over infection, and the association between TB and HIV 79% endorsement. Although these did not meet our threshold of 80%, this does indicate some additional limited variance. With these exceptions, items performed well with relatively adequate variability and are free of extreme floor and ceiling effects. All values of skewness fall between acceptable values of –3 to 3 [20] and demonstrate strong reliability with all alphas measuring greater than 0.9 (See Table 3 for individual item analysis).

**Table 3:** Individual item analysis.

Instrument Item	Mean (0–3)	SD	Skewness	Item-test correlation	Item-rest correlation	Inter-item correlation	Alpha
All items begin with the prefix "Some people who have TB. . ."							
. . .Feel hurt of how others react to knowing they have TB	2.14	0.90	-0.88	0.6549	0.5803	0.4767	0.9092
. . .Lose friends when they share with them they have TB	1.89	1.08	-0.48	0.7809	0.7286	0.457	0.9025
. . .Feel alone	1.82	1.05	-0.48	0.8238	0.7803	0.4503	0.9001
. . .Keep their distance from others to avoid spreading TB germs	2.3	0.87	-1.19	0.6147	0.534	0.483	0.9113
. . .Are afraid to tell those outside their family that they have TB	2.0	0.96	-0.60	0.7025	0.6357	0.4692	0.9068
. . .Are afraid of going to TB clinics because other people may see them there	1.60	1.12	-0.17	0.685	0.6152	0.472	0.9077
. . .Are afraid to tell others that they have TB because others may think that they also have HIV	1.91	1.06	-0.57	0.7806	0.7281	0.4571	0.9025
. . .Feel guilty because their family has the burden of caring for them	1.66	1.06	-0.40	0.7834	0.7315	0.4566	0.9024
. . .Will choose carefully who they tell about having TB	2.23	0.80	-1.06	0.627	0.5481	0.481	0.9107
. . .Feel guilty for getting TB because of their smoking, drinking, or other careless behaviors	2.04	0.91	-0.80	0.7243	0.6613	0.4658	0.9056
. . .Are worried about Having HIV	1.98	0.88	-0.77	0.7349	0.6737	0.4642	0.905
. . .Are afraid to tell their family that they have TB	1.44	1.10	-0.13	0.6716	0.5996	0.4741	0.9084
Test Scale						0.4673	0.9132

SD, standard deviation

During factor analysis, the items maintained adequate factor structure. All items loaded onto one factor, consistent with the original validation, with loadings ranging from 0.559–0.816. Factor loadings and communalities are available in Table 4. Communalities, or the proportion of variance in an item explained by the factor, were less robust with ranges between 0.3405 and 0.6653. Four of the individual items demonstrated communalities <0.4 indicating that there are other factors at play that may explain the variance noted within the item.

**Table 4:** Factor loadings and communality.

Item	Factor loading	Communality	SMC
. . .Feel hurt of how others react to knowing they have TB	0.6051	0.3661	0.5400
. . .Lose friends when they share with them they have TB	0.7645	0.5844	0.7965
. . .Feel alone	0.8156	0.6653	0.7564
. . .Keep their distance from others to avoid spreading TB germs	0.5591	0.3126	0.5797
. . .Are afraid to tell those outside their family that they have TB	0.6643	0.4412	0.5204
. . .Are afraid of going to TB clinics because other people may see them there	0.6476	0.4194	0.6036
. . .Are afraid to tell others that they have TB because others may think that they also have HIV	0.7720	0.5959	0.7012
. . .Feel guilty because their family has the burden of caring for them	0.7661	0.5869	0.7248
. . .Will choose carefully who they tell about having TB	0.5835	0.3405	0.5642
. . .Feel guilty for getting TB because of their smoking, drinking, or other careless behaviors	0.6932	0.4805	0.6807
. . .Are worried about Having HIV	0.7077	0.5008	0.6710
. . .Are afraid to tell their family that they have TB	0.6301	0.3971	0.5179

SMC, squared multiple correlation

### Qualitative data

During the qualitative analysis, we identified several aspects of TB stigma that were most salient from those who endorsed it: (1) TB stigma negatively impacted self-concept reducing self-actualization, (2) anticipated status loss due to TB and (3) TB violated occupational and social roles within the community. These themes are not reflected in the *Patient Perspectives Towards Tuberculosis* but were central to the lived experiences of TB in this sample when they entered RR-TB treatment.

#### Theme 1—TB stigma reduces self concept and inhibits self-actualization

Consistent with prior theory and the body of TB stigma literature, participants explained that TB had reduced their ability to self-actualize or reach their potential. Many of these feelings were the result of disease-related fatalism. Because many participants saw death as a likely outcome, they lost motivation, giving up on goals and feeling undeserving of love, time, and investment. Reflecting on her TB diagnosis, one participant recounts “*I thought it was the end of the road for me*, *I thought I will die*” (LEAP401). Another participant describes the impact of TB on their self-concept and outward presentation, “*I have a low self-esteem now and I always feel like everywhere I am*, *people can tell that I am sick*,” (LEAP117). In this context, RR-TB disease and its potential for fatality eroded self-concept and future-oriented hope. Participants put their goals and plans indefinitely on hold, which both led to and arose from feelings of worthlessness and a sense of languish and inevitable death. Another participant discussed the stagnation they felt after receiving a diagnosis of RR-TB, *“I don’t like the situation I am in*, *I feel bad*, *and I blame myself*. *I want to get passed it and accept but it is difficult*. *I feel like my life is stuck*,*” (LEAP115)*. While this perception of fatality changed over time, it was a theme interwoven throughout interviews.

#### Theme 2—Anticipated status loss due to TB

Participants feared gossip, jokes at their expense, and judgement all of which undermined social status in the local community. As a result, participants carefully guarded their TB status, only disclosing to trusted family or confidants. Beyond non-disclosure, protection against status loss included hiding medications, and self-isolation to avoid involuntary disclosure based on physical appearance. One participant explained changes in her physical appearance would impact her social presentation, *“Now that I have TB*, *people who used to know me as a full-figured lady*, *will now look down on me because of the weight I lost*, *and people will no longer treat me the same way they used to” (LEAP109)*. Physical changes due to either TB-disease or TB treatment such as weight loss and skin darkening were of particular concern. Another participant expands upon this sentiment, *“Some even ask what [is] wrong with me*, *why am I losing so much weight*?.*It was hard for me to even look at myself in the mirror*,*” (LEAP413)*. Many participants discussed the importance of dignity and respect in Zulu culture, that a man’s status in the community was predicated on his behavior and his name. In this cultural context, TB status undermined respect and community status. Echoing this, one participant described why he could not disclose, “*I am protecting my dignity because once I tell one person*, *that person will tell other people and they will start talking about me*, *and they will not speak well of me*” (LEAP421).

#### Theme 3—TB interferes with social and occupational roles

In this sample, participants also described the way that TB impacted their ability to fully participate in society as they once had. This led to disruptions in work, relationships and prospects for marriage and developmental milestones. One participant explains how developing TB disease was incongruent with his occupation, “*The type of job I am doing*, *I found it very embarrassing to be sick with TB as a nurse*” (LEAP121). He goes on to explain that this eventually led to him leaving care during his first TB diagnosis. Other participants explained how TB stigma prevented full social inclusion, *“They isolate you and do not include you in the things that happen in the community*, *they don’t want to be seen with you*,*” (LEAP412)*. In extreme cases, participants described how RR-TB led to stigma even in familial life, where several participants were pressured to leave their homes.

### Mixed data

Triangulating the quantitative and qualitative data, many participants who scored in the high range (>25/50) denied qualitatively that they experienced, perceived, or internalized stigma. During the cognitive interviews, participants reported confusion about item wording that caused them to endorse items that were not consistent with their lived experience. This was well characterized by a participant who remarked, “*When you asked me that question*, *I was responding per the community I live at now*, *not the community I am originally from*, *and I was not responding on my [own] behalf*. *It was on the behalf of the experience of other people*” (LEAP106). Several participants asked clarifying questions about the stigma items, unsure whether to answer based on their own experiences, the experiences of others they knew with TB, or to respond in the affirmative to hypothetical scenarios. Table 5 below provides exemplars of participant responses, quantitatively endorsing items that they deny qualitatively.

**Table 5:** Triangulation of quantitative stigma responses to qualitative experiences.

Study ID	Stigma response	Corresponding quote
LEAP115	Some people who have TB will choose carefully who they tell about having TB - **Agree**	"I don’t have a problem with people with TB, but people still feel the need to hide it, even my friend hid it from me. He only disclosed to me when I told him that I have TB. It shocked me that I have been friends with him, but he doesn’t trust me enough to tell me about his status, but I also understood that some people still feel ashamed of TB, and it is not easy for them to disclose"
LEAP408	Some people who have TB feel hurt of how others react to knowing they have TB. **- Strongly Agree**	"No, I haven’t heard that people experienced stigma from other people, unless it was internalized stigma. Especially because of TB, maybe people experienced Stigma because of HIV, but not because of TB."
LEAP401	Some people who have TB are afraid to tell those outside their family that they have TB.–**Strongly Agree** Some people who have TB lose friends when they share with them they have TB.—**Strongly Agree**	**Participant**: Yes, a lot of people know about my [TB] status, it doesn’t help to not talk because I will suffer alone. **Interviewer**: Okey, so how did they react when you told them? **Participant**: No, there is no change, they are still the same
LEAP410	Some people who have TB lose friends when they share with them they have TB.—**Agree**	**Participant**: I have told some people [my TB status], just some of the people I am close to like my friends. **Interviewer**: How did they react when you told them? **Participant**: They did not show any problems, maybe it is because I explained a bit about TB to them and now, they are the ones encouraging me to never miss my treatment.
LEAP213	Some people who have TB keep their distance from others to avoid spreading TB germs.—**Agree**	**Participant**: Yeah, they [my friends] do visit at my house. And then I bring them chairs and stools and we stay outside and we chat as usual.

Further exploring variance and calibration of the scale, there were several participants with the same stigma scores who described vastly different experiences and feelings about stigma and shame related to TB. Two participants, LEAP306 and LEAP303 were demographically similar, both single women in their 30s, both educated and employed in urban areas. At baseline they both endorsed the same stigma scores, 39/50, and both strongly agreed with items about TB status disclosure. However, these participants qualitatively described different perceptions and feelings about stigma and shame related to TB. LEAP306 shared her TB status with all of her coworkers shortly after diagnosis out of a desire for them to test. She remarks, *“I didn’t see anything to hide in that*.*”* In sharp contrast LEAP303 explains her thoughts on disclosure, *“There are things that you can’t share with anyone*. *Even if they are your friends*, *if they are not your friends ‘like that’*, *you can’t share your personal things with them*.*”* These participants offered the same responses to stigma items about disclosure despite vastly different thoughts and perspectives.

## Discussion

The purpose of the original validation study conducted by Van Rie and colleagues was to “identify points of intervention and evaluate the effects of stigma reduction programmes” [9]. However, the scales have never been successfully used towards either of these ends. At baseline, all items loaded onto one factor consistent with the original validation with loadings ranging from 0.559–0.816. However, quantitative scores did not correlate with the qualitative data indicating poor scale performance. Although our team collected longitudinal stigma data, concerns about the underlying validity of the scale have limited the clinical utility of the data. Without an alternative validated instrument, TB stigma remains largely a study of experience rather than the relationships between stigma and TB-related morbidity and mortality.

While content validity has a variety of definitions, at a minimum, it requires a demonstration of domain definition, relevance (often discussed as face-validity), representation (breadth) and adequate test construction [21]. In this sample, we saw that participants were confused about the wording of the items, causing them to focus externally on the experience or theoretical experience of others. This nuance between external and internal foci may seem esoteric but we posit that it undermines the instrument’s validity at its core. The specification of the items as patient facing is in essence its definition. The external focus described by participants demonstrates that the items are an extension of the *Community Perspectives Towards Tuberculosis*, measuring perceived stigma external to the lived experience of TB. This is exemplified by the participant who explains that he is responding on behalf of others in his community, rather than his own experience.

Similarly, content validity asks that we have adequate representation of the theoretical domains of TB stigma. Contemporary stigma theory posits the experience of stigma as a confluence of components, namely labels, stereotypes, separation, status loss and discrimination that together form the larger experience [8, 16]. Using this theory, some people with TB would not only experience stigma but also anticipate negative behavior and even internally ascribe to some of the negative labels and stereotypes about TB. The *Patient Perspectives Toward Tuberculosis* items rely heavily on items specific to disclosure. While disclosure avoidance is a well-documented manifestation of stigma [22–24], disclosure represents only a small portion of the theoretical definition. Looking at real world examples, in this sample, participants were particularly sensitive to missed opportunities, status loss, and violations of social norms that occurred as a result of a TB diagnosis. These culturally salient and theoretically consistent themes are missing from the instrument as it currently stands. Based on our theoretical knowledge of the stigma construct as well as data from this study, status loss is an important facet of stigma and should be represented in a TB stigma scale. Absence of this important domain threatens the content validity of the scale in context. In the original instrument design, Van Rie and colleagues selected items that represented the domains of 1) fear of transmission; 2) values and attitudes associated with TB; 3) TB status disclosure [9]. Status loss and dehumanization are not included though these are the root of negative values and attitudes and from which a fear of disclosure stems.

Measurement is often defined and evaluated by its sensitivity and specificity. In latent variable measurement, an ideal instrument will maximize variation to ascertain meaningful differences in experience. Within the qualitative sample, we saw diversity of both perceptions and experiences; theoretically the range of scores for both the individual items and the overall scale should reflect this variance [21]; however, stigma is dynamic and deeply personal. Any time there is an attempt at generalizability, individual nuance is lost. In their seminal article *Conceptualizing Stigma*, Link and Phelan describe “stigma as a matter of degree” [8]. They explain that the degree of stigma depends on the negative attributes attached to a particular label. Based on our current findings, we posit that the degree of stigma depends on the confluence of intersecting traits, and how well an individual is able to manage these intersecting identities, concealing negative labels and advancing positive attributes [16]. While there are attribute free stigma scales, to our knowledge there are no validated instruments that have successfully confronted the conundrum of intersectional stigma. Some quantitative strategies for confronting the nuance of intersectional stigma are use of interaction terms, multi-level modeling and latent class analysis but each has associated limitations [25]. As a result, some stigma experts continue to endorse mixed-methods research as the most reasonable way forward to confront measurement challenges [25].

Despite the ranges of qualitative responses found in this sample, we saw that several individual items related to disclosure, maintaining physical distance to prevent transmission, and concern about experienced TB stigma lacked expected variance. This may reflect underlying problems with these particular items. For example, the maintenance of physical distance may be an indication of internalized stigma and self-isolation, or may reflect the reality of the currently agreed upon best approaches to public health management of TB. Participants may view isolation as a social obligation out of respect for others rather than a devaluation of self. There are alternative explanations for why this item lacked variability but in the qualitative interviews self-imposed isolation was not discussed as a result of stigma or viewed as a problematic occurrence. Overall, the *Patient Perspectives Towards TB* scale demonstrated high scores despite a large proportion of the qualitative sample unequivocally denying internalized or experienced TB stigma. This indicates that the scale as currently written and within this context does not capture the degree of stigma which is central to the underlying theory of the construct. Without a measure that can capture degree of experience and evaluate predictive validity of clinically relevant outcomes, stigma will remain a largely theoretical construct without meaningful ways for clinicians to intervene.

In this sample, the overlapping TB/HIV stigma items performed adequately but lacked expected variance. A few considerations may aid our understanding of the item performance. Generally, measurement science tries to avoid “double barreled” questions where participants may resonate with one of two facets within an item. The overlapping items in this scale reflect the interrelated nature of the HIV and TB epidemics. HIV is a driver of TB in high-burden countries in sub-Saharan Africa [26], and TB remains the leading cause of death for people with HIV globally [27]. However, the salience of joint TB/HIV items likely varies depending on the HIV prevalence of a given area. While South Africa remains one of the World Health Organization identified high-burden TB and HIV countries [28], all participants in this study were living with both TB and HIV. This may have also led to unpredictable performance of TB stigma items. Future research should explore stigma experienced by people living with TB and/or HIV to consider the impact of TB/HIV co-infection on the scale’s performance.

Looking at the current TB stigma literature, the revalidation of the *Patient Perspectives Towards Tuberculosis* items in Swaziland saw good psychometric performance in the study population but participation was limited to those speaking English. This sampling requirement has notable limitations towards linguistic sensitivity and the possibility of cultural and socioeconomic differences between those who do and do not speak English in Swaziland [12]. Similarly, in this sample we saw adequate performance of the scale during item analysis despite concerning mixed data results. This analysis provides evidence for the need for extensive qualitative exploration of scales beyond quantitative validation when transferring instruments across cultures. To ensure that measures adequately represent the depth and breadth of latent constructs in a given context, instruments must be culturally relevant but also represent key facets of the underlying theoretical construct. Many scales are constructed in English and then translated into local languages. However, this may obscure or shift the original message. In this study, the scales were professionally translated from English into isiZulu and isiXhosa, then local community health workers reviewed each item for clarity. Despite acknowledging that the items were grammatically correct and translated accurately, the community health workers noted that the items were written in an academic tone and contained words outside of the common vernacular. To increase the clarity and contextual representation of the scale, it may be prudent to first construct a scale in a local language and then translate into English or another language for analysis.

### Limitations

Because this instrument appraisal was nested within a randomized clinical trial there is a possibility that the parent intervention had an impact on participants’ level of stigma. To minimize this risk, we enrolled participants from both the intervention and control arm. Video directly observed therapy reduces interactions with the healthcare environment, it may have an impact on stigma. At this time, that impact is unknown. Future studies should investigate the relationship between technology and level of stigma, but to do so, rigorously validated measures of TB stigma are needed. The quantitative sample was small and limited by the parent study sample size, the results of the factor analysis should be interpreted cautiously. Although the study did not aim to validate the Patient Perspectives Towards Tuberculosis, we did not meet the generally accepted threshold of ten participants for every one item and therefore the results. Furthermore, due to varying perspectives on characteristics and traits by culture and context, perspectives on TB stigma may differ in other locations and cultures, generalizability of quantitative findings and transferability of qualitative findings may not extend to other areas outside of KZN. Finally, this sample enrolled people with both HIV and RR-TB who may bear a higher burden of infectious disease stigma which may have impacted the scale’s performance.

## Conclusion

The *Patient Perspectives Towards Tuberculosis* are well validated for measuring stigma internalized, experienced and anticipated by people living with TB. Despite their robust factor structure and frequent use, the scales did not represent the lived experience of tuberculosis stigma in this sample. Cognitive interviewing revealed that participants responded to instrument items based on the theoretical experiences of others rather than their own experiences, perceptions and feelings. Within this sample, the *Patient Perspectives Towards Tuberculosis* are more reflective of community perceptions of TB and do not represent the unique experiences of people living with TB. While statistical psychometrics are essential for evaluating scale performance, instruments constructed without adequate theoretical and experiential underpinnings may lack construct validity despite adequate factor loadings and robust reliability. Updated measures of TB stigma are needed to capture the theory, and local experience of stigma in KZN. It remains to be seen whether an instrument in any context can fully capture the degree and breadth of TB stigma.

## Supporting information

S1 Checklist Inclusivity in global research. (DOCX)

S1 Appendix Standardized education provided to intervention and control participants by the parent study. (DOCX)
