# Supplementary material for: Cognitive dissonance in tuberculosis stigma: A mixed methods analysis of tuberculosis stigma measurement in South Africa
Source: PLOS Glob Public Health. 2024 Nov 20;4(11):e0003932. doi: 10.1371/journal.pgph.0003932 (PMC11578508; doi:10.1371/journal.pgph.0003932)

S1 Appendix. Standardized education provided to intervention and control participants by the parent study


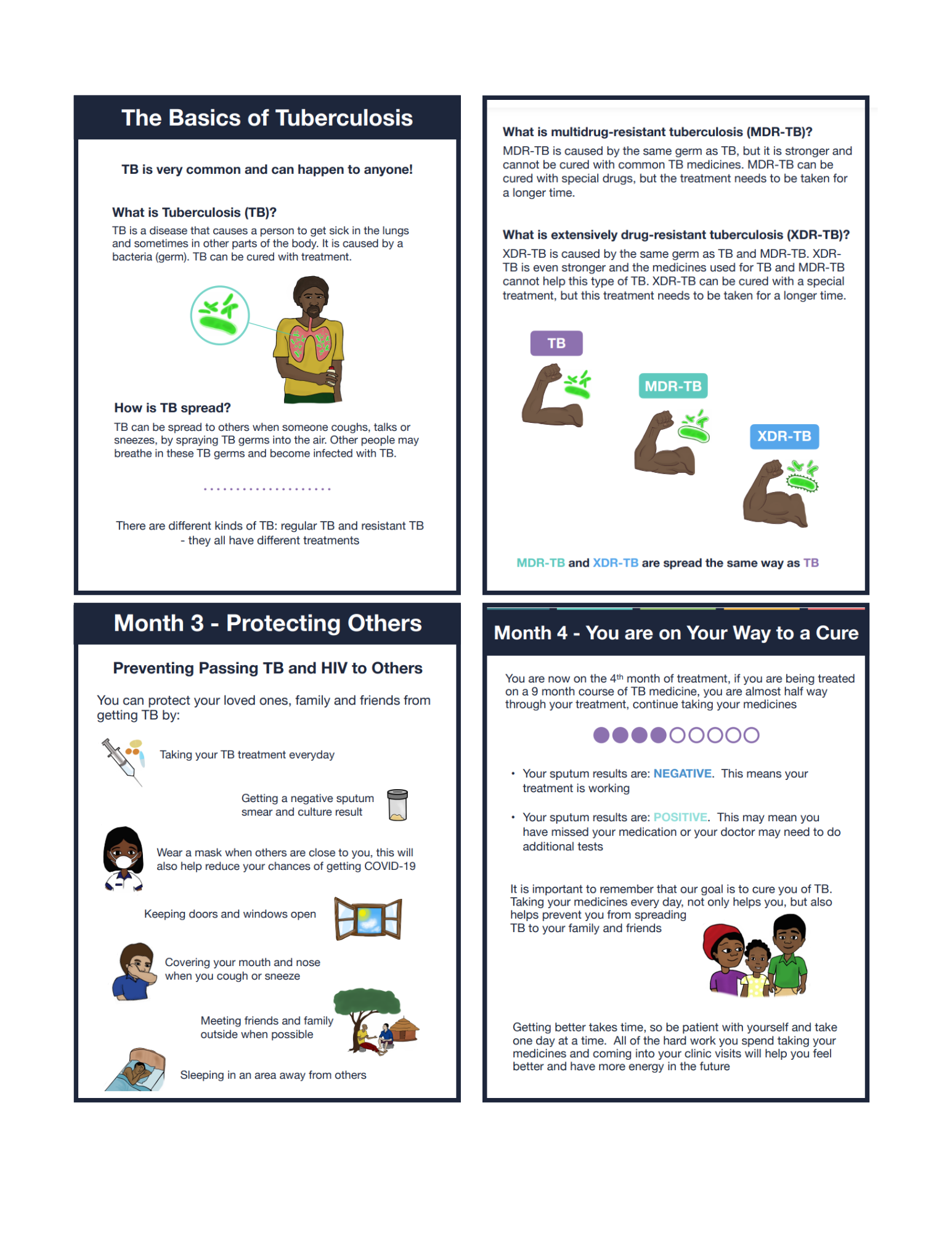

Supplement: S1 Appendix — (DOCX) [file pgph.0003932.s002.docx]
